# Changes in murine anorectum signaling across the life course

**DOI:** 10.1111/nmo.13426

**Published:** 2018-07-30

**Authors:** S. Fidalgo, B. A. Patel, R. N. Ranson, M. J. Saffrey, M. S. Yeoman

**Affiliations:** ^1^ School of Pharmacy and Biomolecular Science Centre for Stress and Age‐Related Disease University of Brighton Brighton UK; ^2^ Department of Applied Sciences Faculty of Health and Life Sciences Northumbria University Newcastle upon Tyne UK; ^3^ School of Life, Health and Chemical Sciences The Open University Milton Keynes UK

**Keywords:** aging, fecal impaction, internal anal sphincter, nitric oxide

## Abstract

**Background:**

Increasing age is associated with an increase in the incidence of chronic constipation and fecal impaction. The contribution of the natural aging process to these conditions is not fully understood. This study examined the effects of increasing age on the function of the murine anorectum.

**Methods:**

The effects of increasing age on cholinergic, nitrergic, and purinergic signaling pathways in the murine anorectum were examined using classical organ bath assays to examine tissue function and electrochemical sensing to determine age‐related changes in nitric oxide and acetylcholine release.

**Key Results:**

Nitrergic relaxation increased between 3 and 6 months, peaked at 12 months and declined in the 18 and 24 months groups. These changes were in part explained by an age‐related decrease in nitric oxide (NO) release. Cholinergic signaling was maintained with age by an increase in acetylcholine (ACh) release and a compensatory decrease in cholinesterase activity. Age‐related changes in purinergic relaxation were qualitatively similar to nitrergic relaxation although the relaxations were much smaller. Increasing age did not alter the response of the anorectum smooth muscle to exogenously applied ACh, ATP, sodium nitroprusside or KCl. Similarly, there was no change in basal tension developed by the anorectum.

**Conclusions and Inferences:**

The decrease in nitrergic signaling with increasing age may contribute to the age‐related fecal impaction and constipation previously described in this model by partially obstructing defecation.

**Key Points**

- The effects of age on excitatory and inhibitory motor neuron signaling in the anorectum are not well understood.
- Aging reduces nitrergic relaxation by decreasing NO release. Cholinergic signaling is maintained through a reduction in cholinesterase activity/expression and an increase in acetylcholine release.
- These changes may impair defecation and contribute to fecal impaction.

## 1 INTRODUCTION

The anorectum of the mouse consists of the internal anal sphincter (IAS) and distal most portion of the rectum.^1^ Changes in the functioning of the anorectum with increasing age could contribute to the increased incidence of chronic constipation, fecal impaction and/or fecal incontinence observed in people over 65 years old.^2^, ^3^ Normal defecation is dependent upon coordinated involuntary actions of cells within the IAS and rectum. As in other parts of the gastrointestinal tract (GI tract), contraction and relaxation of the anorectum is regulated by intrinsic enteric and extrinsic autonomic neurons and by the actions of other cell types, including Interstitial cells of Cajal and PDGFRα + cells.^4^, ^5^, ^6^, ^7^, ^8^ Here, we focus on changes in the function of excitatory and inhibitory motor neurons in the mouse anorectum during aging.

Contraction of the mouse IAS and therefore anal pressure is maintained predominantly through the spontaneous contraction of the IAS circular muscle, but also through the activity of excitatory cholinergic motor neurons.^9^, ^10^ Previous work has shown that the young murine IAS contracts following stimulation of excitatory cholinergic neurons, the release of acetylcholine (ACh) and activation of both M2 and M3 receptors which increase intracellular Ca^2+^ and reduce cAMP, respectively, prolonging the activity of myosin light chain kinase.^11^ For appropriate defecation, the young IAS is relaxed by a combination of inhibitory neurotransmitters, including nitric oxide, ATP (or another purine) and vasoactive intestinal peptide (VIP).^5^, ^6^, ^7^ There is some evidence for the co‐localization of the inhibitory neurotransmitters in specific motor neurons in both mouse and human gastrointestinal tissue.^6^, ^12^, ^13^, ^14^ The release of these inhibitory transmitters is frequency dependent with purinergic transmitters released at the lowest firing frequency, followed by NO and then VIP as the neuronal firing frequencies increase.^5^, ^6^, ^7^ In the mouse, release of these neurotransmitters typically hyperpolarizes IAS circular smooth muscle cells, closing voltage gated calcium channels and reducing intracellular calcium.^5^ However, in the rat, purinergic signaling and nitric oxide have differential effects on IAS smooth muscle to affect relaxation.^15^

In other regions of the GI tract, age is associated with a decrease in the numbers of enteric neurons.^16^, ^17^, ^18^ However, we have previously shown that there is no significant age‐related change in the numbers of PGP9.5 immuno‐reactive nerve fibers innervating the IAS smooth muscle.^19^ Despite no overall change in PGP9.5 immunoreactivity, there was an age‐related reduction in the percentage of motor nerve fibers innervating the IAS that are immunoreactive for nNOS and Substance P, although whether these changes compromise function is currently unclear. Interestingly, nerve fibers expressing calretinin (CR), which in mouse small intestine has been reported to be expressed by excitatory smooth muscle motor neurons, interneurons, and some intrinsic sensory neurons, were not changed with age.^19^ An increase in the thickness of the IAS with age has been described in women,^20^, ^21^, ^22^ although this is not associated with changes in basal tone except in women with fecal incontinence.^20^, ^21^

Currently, no studies have examined how the aging process affects physiological signaling in the anorectum and how any observed changes might contribute to constipation, fecal impaction or incontinence. Given the lack of published work in this field this study examined the effects that increasing age has on basal tone and the function of both the excitatory and inhibitory enteric motor neurons that innervate the murine anorectum. The study used a combination of classical organ bath assays and pharmacology to measure IAS function and sensors to detect the overflow of both NO and ACh from the IAS smooth muscle.

## 2 MATERIALS AND METHODS

### 2.1 Ethical approval

All procedures were carried out according to UK Home Office regulations and the Animal Scientific Procedures Act, 1986 and were approved by the University of Brighton Ethics Committee. The manuscript was prepared according to the UK ARRIVE guidelines.

### 2.2 Animals

Eight‐week old male C57BL/6J mice (Harlan, Bicester, UK) were aged in individual ventilated cages under barrier‐reared conditions. Animals were maintained at 19.0 ± 1°C, 55% humidity and fed on a maintenance diet (RM1 (E) 801002 (Special Diet Services) chow). Studies were performed on 3, 6, 12, 18, and 24 month old animals.

### 2.3 Intestinal preparation

Animals were euthanized with CO_2_ and exsanguinated following cervical dislocation. The rectum plus the internal and external anal sphincters were harvested and placed in oxygenated (95% O_2_ and 5% CO_2_) Krebs buffer solution, pH 7.4 (117 mmol L^−1^ NaCl, 4.7 mmol L^−1^ KCl, 2.5 mmol L^−1^ CaCl_2_, 1.2 mmol L^−1^ MgCl_2_, 1.2 mmol L^−1^ NaH_2_PO_4_, 25 mmol L^−1^ NaHCO_3_ and 11 mmol L^−1^ glucose) at 4°C. The external anal sphincter was removed from the preparation and a single ~2 mm wide ring of smooth muscle cut from the distal most end of the remaining tissue and the mucosa carefully removed using micro‐scissors.

### 2.4 Organ bath pharmacology

**Figure 1:**
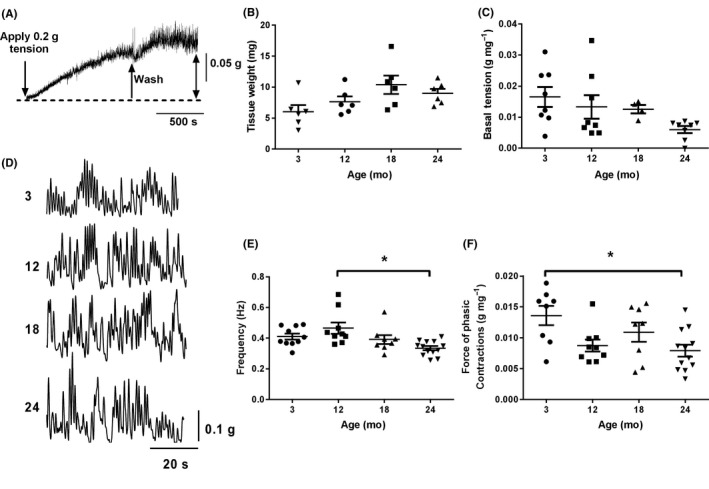
Effects of age on anorectum basal tone and phasic contractions: (A) Isolated anorectum muscle strips generate spontaneous tone when placed under 0.2 g tension in Krebs buffer solution. (B) Age does not affect the wet weight of the anorectum. (C) Basal tension developed by the anorectum is not affected by age. (D) Anorectum muscle strips from 3, 12, 18 and 24 months old animals generate spontaneous phasic contractions in Krebs buffer solution. (E) Frequency of spontaneous phasic contractions is reduced with age *P < 0.05, 12 months vs 24 months. (F) Amplitude of phasic contractions is reduced with age. *P < 0.05, 3 months vs 24 months. Data represent mean ± SEM; n number shown by data points in figure. Sample trace in A is from a 3 month old preparation

Muscle rings were opened by cutting along the mesenteric border and then suspended vertically in a 10 mL organ bath containing Krebs buffer solution at 37°C. Tissues were initially put under 0.2 g tension and allowed to equilibrate for 45 minutes with the Krebs buffer solution changed every 15 minutes. During this time, the tissue developed its own tension which typically plateaued after 30 minutes. Tissues that failed to develop their own tension were excluded from the analysis. Basal tension was the tone developed by the tissue once it had been put under 0.2 g tension and was measured to the mean lower limit of the spontaneous phasic contractions observed during the last minute of the 45 minute equilibration period (double‐ended arrow on Figure 1A). The amplitude and frequency of the phasic contractions were also analyzed using the last 1 minute of recording of the initial 45 minute equilibration period. The amplitude and frequency of the contraction were analyzed using the “cyclic frequency” and “cyclic height” functions in Labchart 7. Tissues were stimulated electrically by suspending them between two platinum wires and passing current pulses (70 V, 0.3 ms pulse width; 0.1‐5 Hz) for 30 seconds every 5 minutes. To confirm that the field stimulation was targeting the nerve fibers in the muscle and was not directly stimulating the smooth muscle we ensured that no response occurred to EFS‐stimulation in the presence of 400 nmol L^−1^ tetrodotoxin (TTx). Tissue responses were also evoked by 10 or 30 μmol L^−1^ nicotine applied to the tissue for 20 seconds every 10 minutes and tissues were washed after each application. Two concentrations of nicotine were chosen as preliminary results suggested that 10 μmol L^−1^ nicotine preferentially stimulated purinergic transmission over nitrergic transmission and we wanted to confirm that this was not solely due to the strength of the nicotinic stimulation.

To isolate the contractile responses muscle strips were incubated with 10 μmol L^−1^ guanethidine (norepinephrine antagonist), 100 μmol L^−1^ L‐N^G^‐Nitroarginine; N^G^‐nitro‐L‐Arginine (L‐NNA; nitric oxide synthase inhibitor) and 1 μmol L^−1^ MRS2500 (P_2_Y_1_ purinergic receptor antagonist). Relaxations were isolated by incubating the tissue in 10 μmol L^−1^ guanethidine, 1 μmol L^−1^ scopolamine (muscarinic antagonist), 1 μmol L^−1^ RP67580 (NK_1_ tachykinin receptor antagonist) and 1 μmol L^−1^ GR159897 (NK_2_ tachykinin receptor antagonist). All antagonists were left in contact with the tissue for 20 minutes to ensure complete block of their respective receptors.

Data were recorded onto computer using ADI instruments bridge amplifier and Labchart 7 software (ADI Instruments, Oxford, UK). Responses were plotted as the integral of the response minus the integral of an equivalent time period immediately preceding the stimulus. All data were normalized to the wet weight of the tissue so data are presented as g.s mg^−1^. Data on the weight of tissue, basal tension, and the frequency and amplitude of phasic contractions were analyzed using a one way ANOVA followed by a Tukey's post hoc test.

### 2.5 Amperometric monitoring of transmitter overflow

#### 2.5.1 Nitric Oxide overflow

Muscle strips were perfused with warm (37°C) Krebs buffer solution at a flow rate of 2 mL min^−1^. Amperometric measurements were made using a 40 μm boron‐doped diamond (BDD) microelectrode as previously described 23 and a stainless steel wire served as the counter electrode and a “no leak” Ag|AgCl electrode (EE009, ESA Biosciences Inc., Sunnyvale, CA) was used as the reference electrode. Amperometric measurements were carried out using a BioStat^™^ multimode potentiostat (ESA Biosciences). The BDD microelectrode was reproducibly positioned 100 μm from the smooth muscle using a micromanipulator (Model 25033, Fine Scientific Tools, North Vancouver, BC). The electrode was held at a detection potential of +1.0 V vs Ag|AgCl, which was sufficient to oxidize NO at a mass‐transfer limited rate. To evoke NO release from the myenteric plexus, a local superfusion pipette was placed within 100 μm of the tissue, and the tissue superfused with 10 μmol L^−1^ nicotine for 20 seconds (Sigma‐Aldrich, UK) to depolarize enteric neurons in the presence or absence of the inhibitors L‐NNA (100 μmol L^−1^) and hexamethonium (10 μmol L^−1^). Triplicate responses were taken from a range of locations across the smooth muscle and averaged to produce a single oxidation current for each tissue. These values were converted to equivalent concentrations of NO using a calibration curve constructed using pure NO gas.^24^ Data were statistically analyzed using a one‐way ANOVA, followed by a Tukey's multiple comparison test.

#### 2.5.2 Acetylcholine (ACh) release

Muscle strips were pinned out in a Sylgard‐lined flow cell bath perfused continuously at 2 mL min^−1^ with warmed (37°C) Krebs buffer solution containing 100 μmol L^−1^ L‐NNA and 10 μmol L^−1^ guanethidine. To detect ACh, amperometric biosensors for detection of ACh and choline (CHO) were utilized as the working electrodes (Sarrisa Biomedical Ltd, Warwick, UK). The ACh and CHO biosensors were held at +500 mV vs Ag|AgCl reference electrode. A stainless‐steel rod served as the counter electrode. A Biostat potentiostat was used to apply the voltage and monitor the current from the working electrode. The biosensors were sequentially placed on the surface of the muscle for each pharmacological treatment. To stimulate release of ACh, 10 μmol L^−1^ veratridine (voltage‐gated Na^+^ channel opener) was perfused for 20 seconds within 1 mm of the muscle strip using a glass capillary at a flow rate of 0.5 mL min^−1^. Control measurements were made 3‐4 times followed by similar recordings in the presence of 1 μmol L^−1^ physostigmine (cholinesterase inhibitor). To calculate the amount of ACh produced, the difference in the current signal obtained by the ACh and CHO biosensors was obtained. The recordings in the presence and absence of physostigmine were each averaged to give a single value for each tissue. Data were then compared using a two‐way ANOVA with age x physotigmine as the two variables, followed by a Tukey multiple comparison post hoc test.

### 2.6 Electrophysiological recordings of anorectum smooth muscle cells

Muscle strips were isolated as described earlier and pinned in a 2 mL horizontal organ bath between two platinum stimulation electrodes and perfused at 1 mL min^−1^ with Krebs buffer solution. Tissues were stimulated using either single pulses or 10 seconds trains of pulses at 2.5 Hz (70 V, 0.3 ms pulse duration) using a DAM 80 extracellular amplifier. Intracellular recordings were made using glass microelectrodes pulled from borosilicate glass (1.5 mm O.D., Clark Electromedical, UK). Electrodes were filled with 3 M KCl and had typical tip resistances of between 70 and 90 MΩ. Cells were recorded only if they had a resting membrane potential (RMP) more negative than −40 mV and data was sampled at 2 KHz and recorded to computer using an Axoclamp 2 A amplifier and Digidata 1440 A/D converter using Spike vs 5.0 (Cambridge Bioscience U.K.). Data was exported to pClamp 10 (Molecular Devices USA) for further analysis. To analyze the transmitters responsible for the evoked hyperpolarizations muscle strips were perfused sequentially with 1 μmol L^−1^ MRS2500 and 100 μmol L^−1^ L‐NNA allowing 20 minutes equilibration to ensure complete block of each signaling pathway.

### 2.7 Effects of exogenous ACh, sodium nitroprusside (SNP) and KCl on anorectum smooth muscle

ACh was added in increasing concentrations (5 × 10^‐7^‐10^−4^ mol L^−1^), for 1 minute every 10 minutes. The effect of the NO‐donor SNP was examined in muscle strips pre‐contracted with 3 μmol L^−1^ carbachol and dosed cumulatively with concentrations of SNP (10^−7^ M‐10^−4^ M) for 60 seconds each. 30 mmol L^−1^ KCl was added for 1 minute to the tissue to examine whether increasing age affected the ability of the muscle to contract. All experiments were carried out in the presence of 400 nmol L^−1^ TTx to prevent any off‐target effects on the enteric nerves and each agonist was added to a separate set of muscle strips.

Responses were plotted as the integral of the response minus the integral of an equivalent time period immediately preceding the stimulus. All data were normalized to the wet weight of the tissue so data were presented as g.s mg^−1^ tissue. Data were analyzed using a two‐way ANOVA, with age × drug treatment as the two variables. A post hoc Tukey multiple comparison test was used to compare statistical differences between the groups.

*P* < 0.05 was taken as being significant. Data were typically expressed as mean ± SEM except for the electrochemical data which were expressed as mean ± SD.

## 3 RESULTS

### 3.1 Age‐related changes in the endogenous properties of the anorectum

Part of the ability of the anorectum to regulate appropriate defecation is its ability to spontaneously generate a tonic basal tension. Following suspension of the tissue in an organ bath at 37°C under 0.2 g tension, all muscle strips increased the force they generated over the next 45 minute control period (Figure 1A). Muscle wet weight did not change statistically with increasing age (Figure 1B). Normalized basal tension decreased with age although this decrease did not reach significance (Figure 1C).

In addition to generating a tonic basal tension, muscle preparations from all age groups generated spontaneous phasic contractions with occasional short (<5 seconds) periods of quiescence between bouts of phasic activity (Figure 1D). Analysis of the frequency of these contractions and their amplitude showed small, but significant reductions in both parameters with increasing age (Figure 1E‐F).

### 3.2 Components of relaxation in the murine anorectum

**Figure 2:**
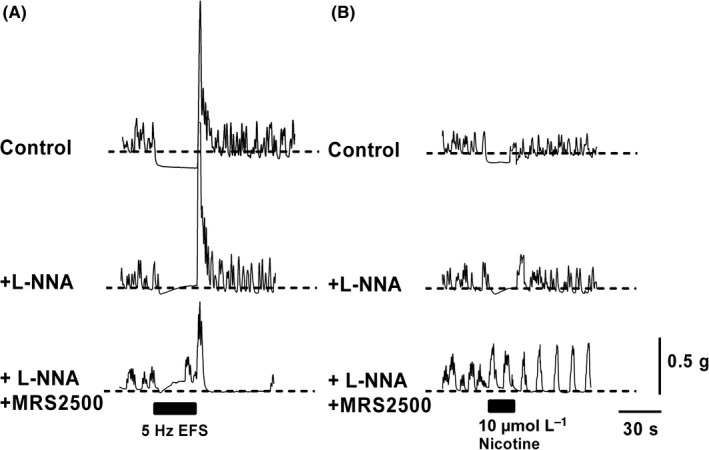
Electrical field stimulation and nicotine‐evoked relaxations in the anorectum: Sample traces from 12 month anorectum recorded under control conditions in the presence of 10 μmol L^−1^ guanethidine, 1 μmol L^−1^ scopolamine and 1 μmol L^−1^ RP67580 and 1 μmol L^−1^ GR159897 to study relaxation. (A) Upper panel. A 30 seconds burst of current pulses (70 V, 0.3 ms pulse duration, 5 Hz) evoked a sustained relaxation and a cessation of phasic activity in the anorectum. Following the cessation of the stimulus, a characteristic rebound contraction was observed, most likely due to increased Ca^2+^ influx through L‐type Ca^2+^ channels. Middle panel, application of L‐NNA blocked the sustained component of the relaxation. Lower panel, MRS 2500 blocked the majority of the remaining transient relaxation. (B) Upper panel, 10 μmol L^−1^ nicotine applied for 20 second evoked a sustained relaxation of the anorectum bathed in Krebs buffer solution. Middle panel, application of L‐NNA blocked the sustained component of the relaxation. Lower panel, MRS 2500 blocked the majority of the remaining transient relaxation and unmasked a contractile component that was not blocked by scopolamine, or the NK1/2 antagonists

Inhibitory nerves innervating the anorectum relax the sphincter allowing for appropriate defecation. The nerves supplying the anorectum can be stimulated to release their neurotransmitters either by electrical field stimulation (EFS) or using nicotine (Figure 2A‐B). In the presence of guanethidine, scopolamine and selective NK1/2 blockers, application of a 30 seconds train of stimuli at 5 Hz across the muscle strip drove a sustained relaxation and halted phasic contractions of the smooth muscle (Figure 2A). Application of 100 μmol L^−1^ L‐NNA to block nitrergic signaling blocked the sustained component of the relaxation leaving a transient relaxation. Further addition of 1 μmol L^−1^ MRS2500 to block purinergic signaling almost completely removed the relaxation and unmasked a delayed contractile component to the evoked response. Application of nicotine for 20 seconds also evoked a sustained relaxation and halted phasic contractions in the smooth muscle (Figure 2B). Application of L‐NNA again blocked the sustained component of the response leaving a transient relaxation. Further addition of MRS 2500 blocked the remaining relaxation and exposed a small component of contraction that was not blocked by a combination of scopolamine and the NK1/2 blockers.

### 3.3 Effects of age on the L‐NNA‐sensitive component of the relaxation

**Figure 3:**
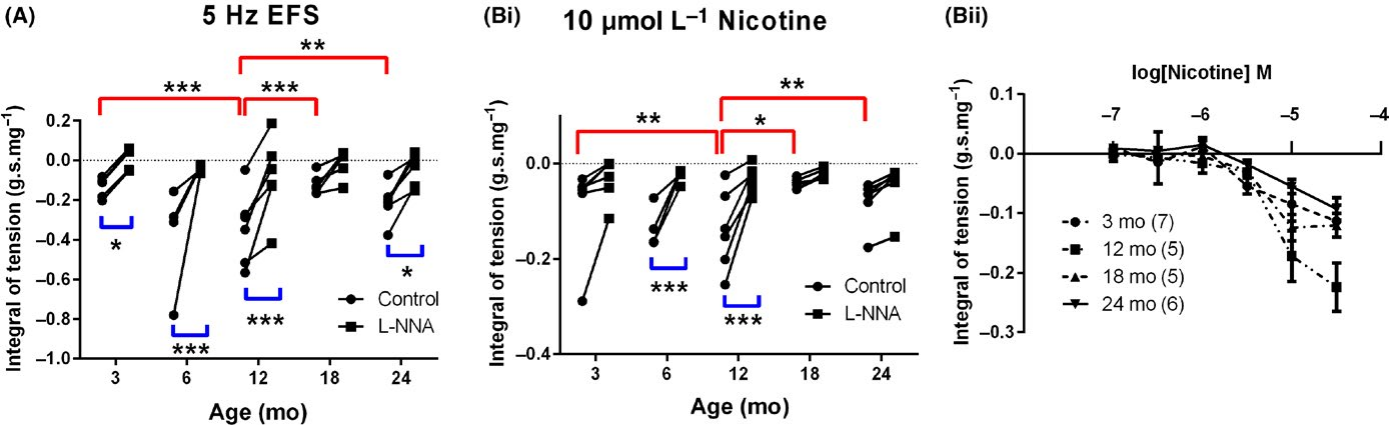
Lifecourse changes in nitrergic relaxation in the anorectum. EFS‐evoked (A) and nicotine‐evoked (Bi) nitrergic relaxation increases between 3 and 12 months and then decreases at 18 and 24 months. (Bii) Nicotine evoked dose‐dependent relaxations in anorectum smooth muscle are unaffected by age. The black trend lines show how relaxation in a specific muscle strip is affected by L‐NNA. Red lines in A and B represent significant age effects between control responses. Blue lines represent significant drug (L‐NNA) effects within a given age group. *P < 0.05; **P < 0.01, ***P < 0.001. Values are mean ± SEM. In part A and Bi, n = 6 for 3, 12, 18, and 24 month groups and n = 4 for 6 month group. In part Bii, the numbers in brackets represents n number for part Bii

The effects of increasing age on the ability of both EFS and nicotine to evoke muscle relaxation are shown in Figure 3. There was a significant increase in the integral of EFS‐evoked relaxations between 3 and 12 months which declined back to 3 month levels at 18 and 24 months (Figure 3A, red lines). A significant L‐NNA‐sensitive component to the EFS‐evoked relaxation was observed in the 3, 6, 12 and 24 month groups (Figure 3A; blue lines).

The integral of the nicotine evoked relaxations also increased between 3 and 12 months declining back to 3 month values in both the 18 and 24 month age groups (Figure 3Bi; red lines). Application of L‐NNA significantly reduced nicotine‐evoked relaxations in the 6 and 12 month age groups (Figure 3Bi; blue lines).

Concentration response curves to nicotine showed no age‐related changes in the threshold concentration required to evoke a measurable relaxation, nor any significant difference of age when compared with a two‐way ANOVA (Figure 3Bii).

### 3.4 Effects of age on nitric oxide overflow from anorectum smooth muscle

**Figure 4:**
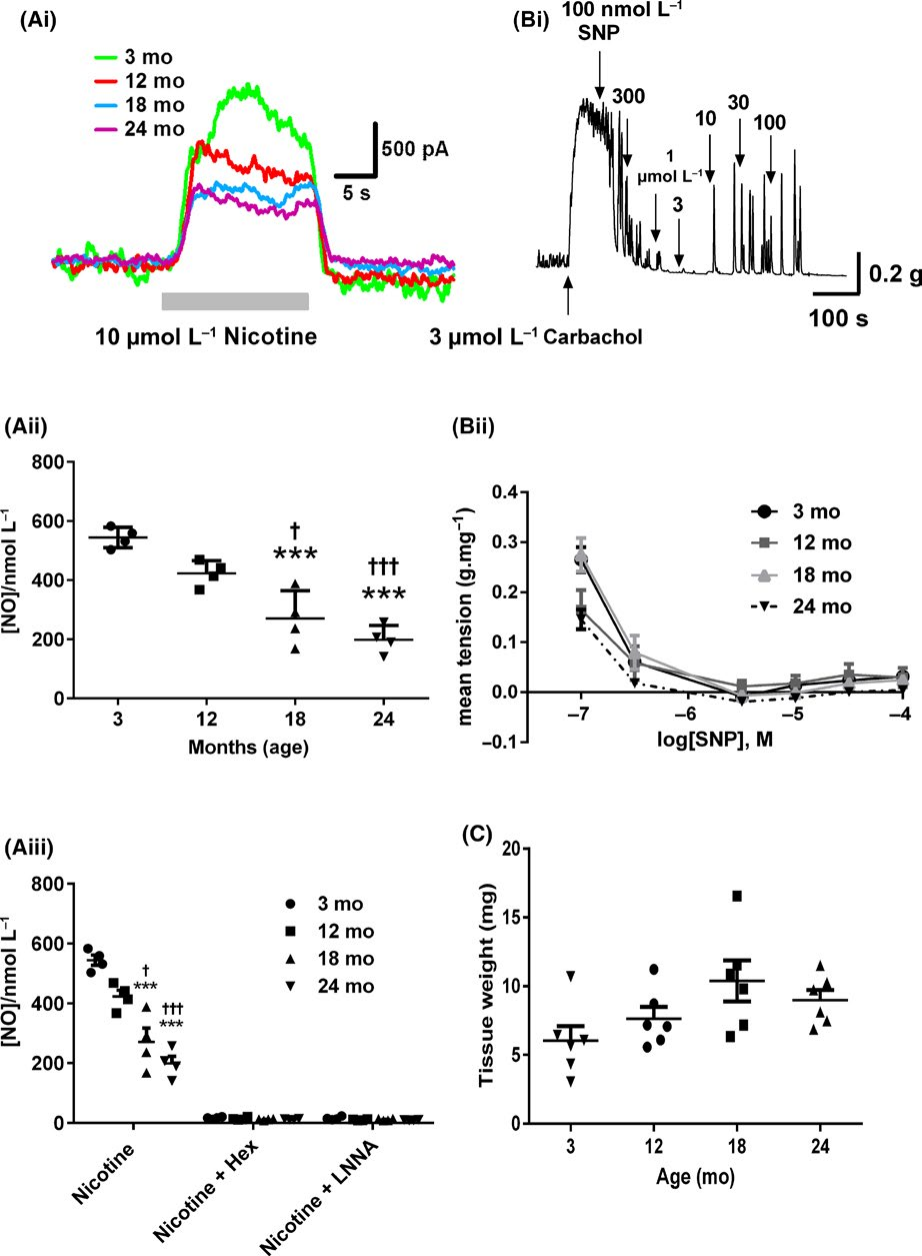
Age‐related changes in nitrergic signalling in the anorectum. (Ai) Typical NO oxidation current traces evoked by nicotine (perfused for the duration of the gray bar) in 3, 12, 18, and 24 month anorectum. (Aii) NO overflow is reduced with increasing age. (Aiii) Nicotine‐evoked NO overflow is blocked by hexamethonium and L‐NNA. (Bi) Typical trace from a 3 month anorectum showing SNP‐evoked relaxations in a carbachol precontracted muscle strip. (Bii) SNP‐evoked relaxations are not affected by age. (C) KCl‐evoked contractions are not affected by age. ***P < 0.001 vs 3 month, ^††^ *P* < 0.01 vs 12 month. Data in A represent mean ± SD; data in B and C represent mean ± SEM. N = 4 for each group

We next examined whether the age‐related changes in the L‐NNA‐sensitive component of the relaxation were due to changes in nitric oxide release or the sensitivity of the smooth muscle cells to SNP. Given that nicotine significantly increased relaxations between 3 and 12 months we chose to examine NO overflow in 3, 12, 18, and 24 month preparations. Nicotine‐evoked NO overflow showed a gradual decline with increasing age, with a significant decrease being observed in the 18 and 24 month age groups vs both the 3 and 12 month groups (Figure 4Ai/ii). Nicotine‐evoked release was completely inhibited in all age groups following the application of either 10 μmol L^−1^ hexamethonium or 100 μmol L^−1^ L‐NNA (Figure 4Aiii).

### 3.5 Effects of age on the response of the anorectum muscle to SNP and KCl

To examine the sensitivity of the anorectum to NO, we applied increasing concentrations of the NO‐donor SNP to the tissue that had been precontracted with carbachol (Figure 4Bi). Dose‐response curves to increasing concentrations of SNP showed no significant effect of age (Figure 4Bii). KCl‐evoked contractions were also not affected by increasing age (Figure 4C).

### 3.6 Effects of age on the MRS 2500‐sensitive component of the relaxation

**Figure 5:**
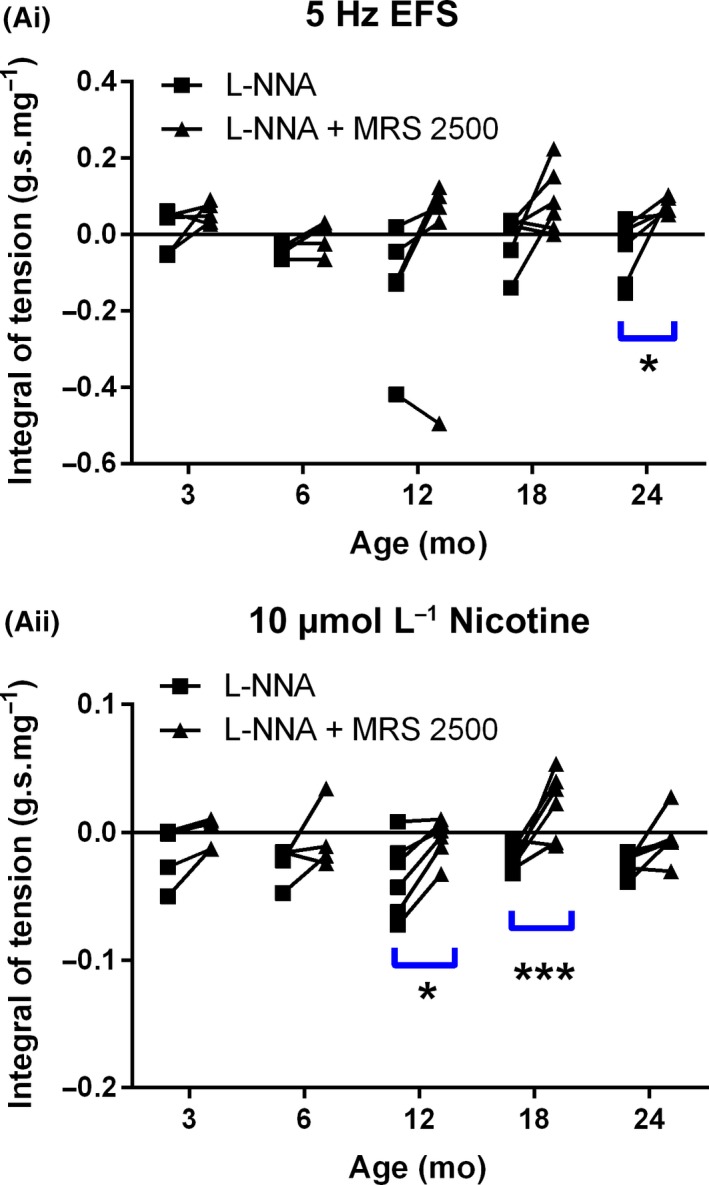
Age‐related changes in purinergic relaxation in the anorectum. Effects of age on EFS‐ (Ai) and nicotine‐ (Aii) evoked purinergic relaxations. The black trend lines show how relaxation in a specific muscle strip is affected by MRS2500. Blue lines represent significant drug (MRS2500) effects within a given age group. *P < 0.05; ***P < 0.001 vs respective controls. In parts Ai/Aii, n = 6 for 3, 12, 18, and 24 month groups and n = 4 for 6 month group. Data represent mean ± SEM

In L‐NNA treated preparations there was no significant difference in size of the EFS or nicotine‐evoked relaxations with increasing age (Figure 5A). Although the P_2_Y_1_ receptor antagonist MRS‐2500 reduced the amplitude of the EFS‐evoked relaxation in muscle strips from all age groups this only reached significance in the 24 month group (Figure 5Ai). Significant nicotine‐evoked MRS 2500‐sensitive responses were observed in both the 12 and 18 month groups (Figure 5Aii).

### 3.7 Effects of age on spontaneous and evoked inhibitory junctional potentials (IJPs)

**Figure 6:**
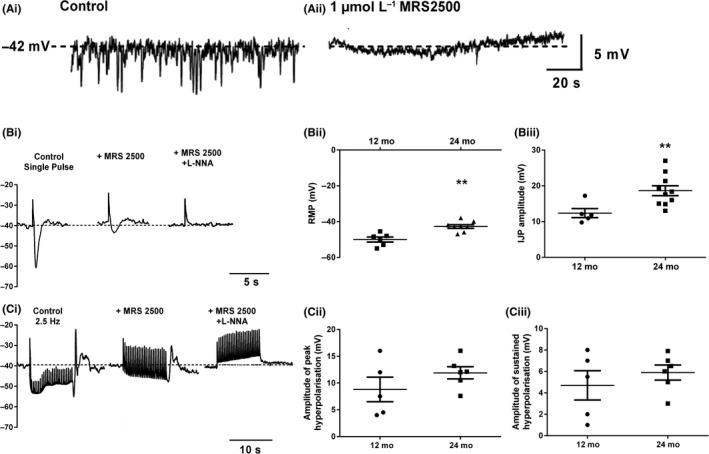
Effects of age on spontaneous and evoked IJPs in muscle fibers from the anorectum. (Ai) Typical trace of an intracellular recording from a 12 month muscle cell showing spontaneous miniature IJPs. (Aii) Spontaneous miniature IJPs are blocked by MRS 2500. Dotted line represents the resting membrane potential of the muscle cell. (Bi) Typical traces from a 12 month muscle cell showing IJPs evoked by a single current pulse under control conditions and in the presence of MRS 2500 and MRS 2500 + LNNA. (Bii) Increasing age caused a depolarization of the smooth muscle cell resting membrane potential. (Biii) Increasing age increased the amplitude of current‐evoked IJPs. (Ci) Trains of current pulses (2.5 Hz) evoked a sustained hyperpolarization of the smooth muscle under control conditions and in the presence of MRS 2500 and MRS2500 + L‐NNA. *N.B. Stimulus artefacts have been artificially reduced to show the slow electrical response more clearly. Fast upward vertical deflections are the remaining stimulus artefacts*. Age did not affect the amplitude of the peak (Cii) or sustained (Ciii) hyperpolarization. **P < 0.01. Data represent mean ± SEM. N number represented by number of symbols on figures

The effects of age on the properties of both spontaneous and evoked IJPs recorded from anorectum smooth muscle cells were examined in the presence of scopolamine, guanethidine and nifedipine. We focused on the 12 and 24 month age groups as 12 months represented the peak of the EFS/nicotine‐evoked relaxations in the IAS and 24 months, the time point where we had previously demonstrated clear signs of fecal impaction and a constipation phenotype.^25^ Intracellular recordings from single muscle cells showed spontaneous miniature inhibitory junction potentials (mIJPs) superimposed on the resting membrane potential (RMP) (Figure 6Ai). These mIJPs were completely blocked following the addition of MRS2500 (Figure 6Aii). The amplitude and frequency of these spontaneous mIJPs was not altered with increasing age (data not shown).

Individual current pulses gave rise to well‐defined IJPs that caused a rapid hyperpolarization of the RMP followed by a slower decay phase (Figure 6Bi; left‐hand panel). Following the end of the stimulus the membrane potential transiently overshot the RMP depolarizing the muscle cell before returning to baseline. The RMP of the muscle cells was more depolarized with age (Figure 6Bii) and there was a consequential increase in the amplitude of the evoked IJP (Figure 6Biii). Addition of MRS 2500 blocked the majority of the IJP in both age groups (73.5% and 83.3% in 12 and 24 months respectively; *P* > 0.05). There was no significant age‐related change in the amplitude of the MRS 2500 or L‐NNA‐sensitive IJPs. Addition of both MRS 2500 and L‐NNA failed to significantly alter the RMP of the smooth muscle cells. Higher frequency stimuli (2.5 Hz) evoked longer lasting hyperpolarizations of the membrane potential, that consisted of a transient rapid decrease in the membrane potential followed by a sustained hyperpolarization that lasted for the duration of the stimulus (Figure 6 Ci). Following the termination of the burst of stimuli, the cell showed a transient depolarization before returning to the RMP. Application of MRS2500 removed the fast transient component of the response leaving a slowly developing sustained hyperpolarization that was sensitive to L‐NNA (second and third panel Figure 6 Ci). There were no significant age‐related changes in the amplitude of the transient or sustained components of the response (Figure 6Cii/iii).

### 3.8 Effects of age on cholinergic signaling in the murine anorectum

**Figure 7:**
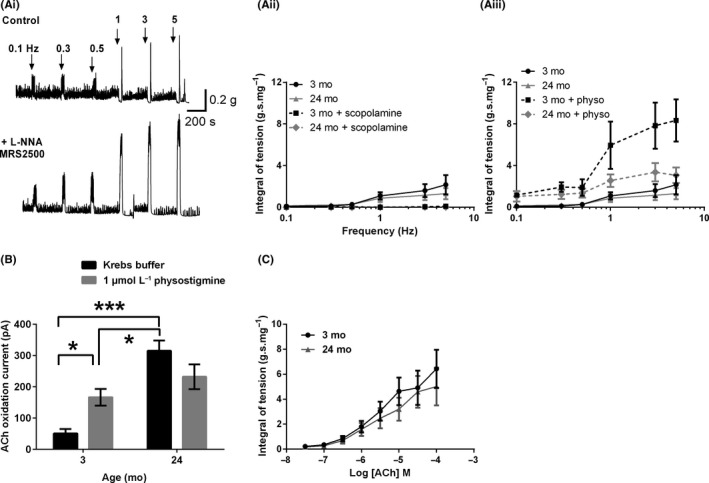
Effects of age on cholinergic signaling in the mouse anorectum. (Ai) Typical trace from a 3 month anorectum showing EFS‐evoked responses recorded in Krebs buffer solution (upper trace) and in Krebs buffer solution containing L‐NNA, guanethidine, and MRS 2500 (lower trace). (Aii) Frequency response curves for 3 and 24 month muscle strips recorded in the presence of guanethidine, L‐NNA and MRS 2500, under control conditions and in the presence of scopolamine. (Aiii) Frequency response curves for 3 and 24 month muscle strips recorded in the presence of guanethidine, L‐NNA and MRS 2500, under control conditions and in the presence physostigmine (B) Veratridine‐evoked ACh oxidation currents recorded under control conditions and in the presence of physostigmine. (C) Exogenous ACh caused dose‐dependent increases in anorectum contractions that were not affected by age. *P < 0.05; ***P < 0.001. Values in B are mean ± SD, n = 3 for each group; values in (Aii/Aiii) and (C) represent mean ± SEM; for parts (Aii/Aiii) and (C) n = 6 per group

EFS‐evoked responses recorded in Krebs buffer solution, yielded an initial contractile response between 0.1 and 0.5 Hz which gave way to a net relaxation at higher frequencies of stimulation (Figure 7Ai; upper panel). In the presence of guanethidine, L‐NNA and MRS 2500, EFS unmasked a frequency‐dependent cholinergic contractile response (Figure 7Ai; lower panel Figure 7Aii). This response increased with increasing frequency (*P* < 0.001) and was almost completely blocked by scopolamine in both age groups tested (*P* < 0.05 both age groups; Figure 7Aii). No differences were seen between the 3 and 24 month control data (*P* > 0.05). Addition of physostigmine caused a marked increase in the contractile response in the young muscle strips (*P *< 0.0001) and a smaller significant effect on the 24 month tissue (*P* < 0.05; Figure 7Aiii). The current flow due to the oxidation of ACh was increased in the old preparations (Figure 7B). This ACh oxidation current was significantly increased in the young following the application of physostigmine, but not in the old tissue (Figure 7B). The ACh oxidation current was significantly lower in young physostigmine treated muscle strips compared to old controls (Figure 7B). Concentration response curves generated following the application of exogenous ACh did not significantly change with age (Figure 7C).

## 4 DISCUSSION

This study investigated the effects of increasing age on the basal tone and cholinergic, purinergic and nitrergic signaling in the mouse anorectum. Our data show conclusively that there is no significant change in basal IAS tone with increasing age. In addition, we demonstrate that both EFS and nicotine‐evoked nitrergic relaxation is reduced with old age and that this impairment appears, at least in part, to be due to a decrease in NO overflow. Cholinergic contractions were maintained with increasing age, due to both an increase in ACh release and a decrease in cholinesterase activity. Age‐related changes in purinergic signaling were more variable and purinergic responses caused weaker relaxations those evoked by NO. However, the effects of age on purinergic signaling were qualitatively similar to the nitrergic responses. Exogenous application of ACh, SNP, ATP, and KCl showed no significant age‐related changes in postsynaptic signaling and muscle responses. The implications of these findings are discussed.

### 4.1 Characterization of age‐related changes in spontaneous anorectum activity

Muscle strips from all age groups generated spontaneous phasic activity superimposed on an increase in basal tone. Although basal tone decreased with age this change was not significant, strongly suggesting that resting tension in the muscle was not affected by 24 months in the mouse. Previous work has shown that the activity of the most distal 1 mm (termed “IAS”) of the anorectum is markedly different from tissue taken 1‐2 mm (termed “distal rectum”) from the anal verge, both in terms of the generation of spontaneous tone and phasic activity.^1^ Further analysis of our data (data not shown) demonstrated that our muscle strips, obtained from all age groups, showed certain similarities with the IAS described by Hall et al. (high basal tone and short periods of quiescence between bouts of spontaneous phasic activity), but also had features of the distal rectum (relatively low frequency of phasic contractions, and low total spontaneous activity when expressed as a % of the maximal KCl‐evoked contraction). Aside from the frequency and amplitude of the phasic contractions which decreased significantly with age, there were no other significant age‐related changes in spontaneous activity of the anorectum.

### 4.2 Changes in murine anorectum nitrergic signaling across the lifecourse

EFS and nicotine‐evoked nitrergic relaxations were slow in onset but were sustained throughout the period of stimulation consistent with observations from previous studies in young animals.^5^, ^9^, ^15^ Importantly, although the amplitude of the relaxations was altered with age the responses kept the characteristic waveform observed in the young. The integral of the relaxations increased between 3‐12 months and then declined at both 18 and 24 months of age. To our knowledge this is the first description of how nitrergic signaling is altered across the life course in any GI tract smooth muscle. Previous studies have shown a decrease in nNOS immunoreactive fibers innervating anorectum circular smooth muscle in both the 18 and 24‐month age groups^19^ and this observation could explain the reduced nitrergic relaxation observed in the older age groups and also the decrease in NO overflow. This reduction in nNOS immunoreactive fibers could represent neurodegeneration in this fiber group consistent with previous observations in the mouse colon^26^ or alternatively could be due to decreases in nNOS expression in these neurons. Results from this study showed a decrease in nicotine‐evoked NO overflow with increasing age. NO overflow decreased from approximately 550 nmol L^−1^ in 3 month tissue to 300 and 200 nmol L^−1^ in 18 and 24 month tissue, respectively. Given that maximal SNP‐evoked relaxations were observed at concentrations around 300‐1000 nmol L^−1^, reductions in NO overflow seen in the 18 and 24‐month age groups could at least partly explain the reduced nitrergic relaxation seen in these age groups. While it is possible that the reduction in nitrergic innervation of the anorectum may explain the age‐related decrease in NO overflow, previous studies looking at the development of nitrergic signals in the guinea pig have shown that decreases in NO detected at the sensor could be due to growth of the tissue with age and a reduction in the density of the nitrergic fibers rather than a reduction in their number.^27^ However, Wang et al. observed no significant change in the width of the anorectum with increasing age,^19^ and the lack of a significant change in anorectum weight with increasing age makes this possibility unlikely.

Although the decrease in NO overflow could potentially explain some of the decrease in nitrergic relaxation seen in the 18 and 24 month age groups it cannot easily explain the increased relaxation observed between 3 and 12 month anorectum. In addition, our observation that there was no significant difference between SNP evoked relaxations in the 3 and 12 month anorectum, nor in the amplitude of nitrergic IJPs or KCl‐evoked contractions, is strongly suggestive that the change in the amplitude of nitrergic relaxations observed between these two groups is not due alterations in postsynaptic signaling. That a qualitatively similar changes in nitrergic‐evoked relaxations was observed using both EFS and nicotine, suggests that the changes that are occurring are independent of the method of stimulation and may reflect age‐related alterations in the electrical excitability of the enteric nerves innervating the anorectum. This is a common phenomenon observed with age in central neurons^28^ and in intrinsic primary afferent neurons (Kunze pers. comm.). Although qualitatively similar, 5 Hz EFS seemed more effective at evoking nitrergic relaxations than applications of both 10 μmol L^−1^ and 30 μmol L^−1^ nicotine (latter data not shown). This might suggest an age‐related change in the nicotinic receptor phenotype as has been described previously in the CNS^29^, ^30^ although age‐related comparisons of the nicotine dose‐response curves showed no significant differences with age, suggesting that this was unlikely. However, we cannot discount the possibility of age‐related changes in nAChR expression on nitrergic neurons in the anorectum.

### 4.3 Changes in purinergic signaling across the lifecourse

In comparison to nitrergic signaling, EFS and nicotine evoked fast but transient purinergic relaxations, consistent with those previously described in mice.^7^ Relaxations were observed in the anorectum from all age‐groups treated with L‐NNA but these responses were small and further addition of MRS2500 to isolate the purinergic component yielded inconsistent results between the two modes of stimulation. Nicotine appeared more efficacious at stimulating purinergic relaxations while EFS was most effective at driving nitrergic relaxations. This difference could be explained by the two different transmitters being present in different populations of nerve terminals as has previously been demonstrated for nNOS and VIP.^12^, ^13^ Burnstock originally described the possibility of co‐transmission of multiple transmitters from a single neuron.^31^ More recently, supporting evidence for the co‐localization of nNOS and ATP has come from studies in the human colon,^32^ however, a recent immunohistochemical study in mice and guinea pigs has shown that there are potentially two distinct classes of inhibitory motor neurons, one purinergic and one nitrergic.^14^ Alternatively, nicotine and EFS may be stimulating the inhibitory motor neurons to fire at different frequencies. The release of purines from the colon has previously been shown to be favoured by low firing frequencies and inhibited by high frequency firing,^32^, ^33^ suggesting that nicotine might favor neuron firing frequencies of less than the 5 Hz EFS. The lack of change in the amplitude of the purinergic IJP between 12 and 24‐month‐old animals, strongly suggested that, like nitrergic signaling, the age‐related changes in purinergic relaxation represented prejunctional changes.

### 4.4 Age‐related changes in cholinergic signaling

Cholinergic signaling, determined by EFS, was maintained between 3 and 24 months. ACh is broken down in the synapse by the actions of cholinesterase. In the presence of physostigmine, a cholinesterase inhibitor, both EFS‐evoked contractions and veratridine‐evoked ACh oxidation signals would be expected to increase as more ACh would reach the target muscle/sensor. This was the case in the young anorectum but the ACh oxidation signal was not significantly altered in the old and the increase in the EFS‐evoked contractions in the presence of physostigmine was greatly attenuated in old anorectum compared to young tissue, strongly suggesting that either the activity or the amount of enzyme present in the synapse was reduced in the old anorectum. We suggest that this decrease would help to maintain excitatory cholinergic transmission and smooth muscle contractility. These changes are different to those observed in the small bowel of the rat, where an age‐related decrease in cholinergic transmission was observed and linked to a potential increase in cholinergic activity. Previous work on aged GI tract has shown an increase in reactive oxygen species in animal models with increasing age,^34^, ^35^, ^36^, ^37^, ^38^, ^39^ although similar changes in the humans have not been so clearly documented.^40^ Oxidative stress has also been demonstrated to reduce cholinesterase activity in a range of tissues providing a potential mechanism for changes observed in this study.^41^, ^42^, ^43^

About 1 μmol L^−1^ physostigmine has been shown to completely block cholinesterase activity in other GI tract smooth muscles. Therefore in the presence of physostigmine the ACh oxidation current is most likely reflective of the amount of ACh released by the tissue. Our observation that the ACh oxidation current is still significantly lower in the young physostigmine treated anorectum compared to the old untreated anorectum, is strongly suggestive that ACh release is also up‐regulated with increasing age providing a second mechanism that contributes to the maintenance of cholinergic signaling with age. While the causes of this proposed increase are not known it is possible that the increased release is a function of an increase in cholinergic nerve terminals, the numbers of cholinergic vesicles, an alteration in choline acetyltransferase activity or an age related increase in intracellular Ca^2+^ which has previously been shown to be elevated with increased age. Postsynaptic signaling pathways, determined by the exogenous application of ACh and KCl‐evoked were unaltered with increasing age.

### 4.5 Proposed impact of age‐related changes in anorectum signaling

The murine anorectum is maintained in a contracted state predominantly by myogenic activity but also through the activity of excitatory cholinergic motor neurons that innervate the circular smooth muscle. Our initial observations on basal myogenic activity show a decrease in basal anorectum tone, however, this change did not reach significance. Previous studies have shown that myogenic activity in the murine anorectum is due to the properties of voltage gated Ca^2+^ channels in the muscle membrane. At the resting membrane potential of the anorectum muscle cells, these currents are active providing an influx of Ca^2+^ which drives the anorectum to contract.^11^ The lack of change in basal tone with increasing age is strongly suggestive that the properties of these channels have not significantly changed with age. Work by Singh et al.^44^ has shown that basal tone in the rat IAS can be increased by low levels of oxidative stress and dramatically reduced as levels of oxidative stress increase above a threshold level.^44^ It is therefore possible that the increase in oxidative stress that has been seen in other smooth muscle systems with increasing age may potentially help maintain tone in the murine anorectum.^45^ Had we left our animals to age past 24 months and had oxidative stress increased further, then we may have observed detrimental effects on anorectum tone which could contribute to fecal incontinence.

Consistent with our observations of a lack of change in anorectum basal tone was our observation that excitatory motor neuron signaling was shown not to change in this study. This lack of a change was explained by an age‐related decrease in the activity/expression of cholinesterase and an increase in ACh release.

For appropriate defecation to take place, the anorectum has to be relaxed through the activation of inhibitory motor neurons that innervate the circular smooth muscle. Nitrergic relaxation increased between 3‐12 months of age and then decreased in 18 and 24 month groups. Most of this decrease in the older age‐groups is caused by a decrease in nitrergic signaling due to a combination of a reduction in the density of nitrergic fibers and NO release. These data strongly suggest that the process of defecation is likely to be impaired in older animals, due to the impaired relaxation of the anorectum. We have previously described that murine aging is associated with fecal impaction and a constipation phenotype with animals producing smaller and harder pellets and a reduction in total fecal output.^31^ Similarly, fecal impaction and a reduction in fecal output have been observed in a model of slow transit constipation, in which a purse string suture was used to restrict fecal output.^46^ The authors showed that these changes were due to an increase in the inflammatory mediator COX‐2 and could be reversed using a COX‐2 antagonist. We too have recently shown that aging is associated with an increase in the pro‐inflammatory mediator TNF‐α and that treatment with a TNF‐α antagonist, etanercept was able to reverse the age‐related changes in pellet output and water content.^47^

In humans, relaxation of the IAS is also driven predominantly by nitrergic signaling. Should similar changes occur in the human IAS then these changes are also likely to impair IAS relaxation in humans and may contribute to age‐related fecal impaction. Contraction in the human IAS is in part regulated by noradrenergic signaling, and while there are reports of reductions in noradrenaline in aged rat GI tissue the functional consequences of these changes are unclear as resting pressures are not altered in aged humans.

In summary, we have shown that the natural aging process reduces evoked relaxation in the murine anorectum in the absence of changes in basal tone or excitatory motor neuron signaling. This change is predominantly due to a decrease in nitrergic signaling and may well contribute to an impairment of fecal output with increasing age and contribute to the fecal impaction and constipation phenotype previously described in this model.^25^

## COMPETING INTERESTS

The authors have no competing interests.

## AUTHOR CONTRIBUTIONS

SF acquisition, analysis of data, and critical revision of the manuscript; BP study concept and design, acquisition, analysis, and interpretation of data, critical revision of the manuscript; MJS critical revision of the manuscript and grant funding; RR critical revision of the manuscript and grant funding; MY study concept and design; data analysis, writing the manuscript; grant funding.

## References

[1] Hall KA , Ward SM , Cobine CA , Keef KD . Spatial organization and coordination of slow waves in the mouse anorectum. J Physiol. 2014;592:3813‐3829. 24951622 10.1113/jphysiol.2014.272542 PMC4192705

[2] Rao SSC , Go JT . Update on the management of constipation in the elderly: new treatment options. Clin Interv Aging. 2010;5:163‐171. 20711435 10.2147/cia.s8100 PMC2920196

[3] Kamm MA . Faecal incontinence. BMJ : British Medical Journal. 1998;316:528‐532. 9501717 10.1136/bmj.316.7130.528 PMC2665648

[4] Cobine CA , Hannah EE , Zhu MH , et al. ANO1 in intramuscular interstitial cells of Cajal plays a key role in the generation of slow waves and tone in the internal anal sphincter. J Physiol. 2017;595:2021‐2041. 28054347 10.1113/JP273618 PMC5350438

[5] Cobine CA , Sotherton AG , Peri LE , Sanders KM , Ward SM , Keef KD . Nitrergic neuromuscular transmission in the mouse internal anal sphincter is accomplished by multiple pathways and postjunctional effector cells. Am J Physiol Gastrointest Liver Physiol. 2014;307:G1057‐G1072. 25301187 10.1152/ajpgi.00331.2014 PMC4254957

[6] Keef KD , Saxton SN , McDowall RA , Kaminski RE , Duffy AM , Cobine CA . Functional role of vasoactive intestinal polypeptide in inhibitory motor innervation in the mouse internal anal sphincter. J Physiol. 2013;591:1489‐1506. 23339175 10.1113/jphysiol.2012.247684 PMC3607168

[7] McDonnell B , Hamilton R , Fong M , Ward SM , Keef KD . Functional evidence for purinergic inhibitory neuromuscular transmission in the mouse internal anal sphincter. Am J Physiol Gastrointest Liver Physiol. 2008;294:G1041‐G1051. 18308858 10.1152/ajpgi.00356.2007

[8] Cobine CA , Hennig GW , Kurahashi M , Sanders KM , Ward SM , Keef KD . Relationship between interstitial cells of Cajal, fibroblast‐like cells and inhibitory motor nerves in the internal anal sphincter. Cell Tissue Res. 2011;344:17‐30. 21337122 10.1007/s00441-011-1138-1 PMC3192126

[9] Duffy AM , Cobine CA , Keef KD . Changes in neuromuscular transmission in the W/W(v) mouse internal anal sphincter. Neurogastroenterol Motil. 2012;24:e41‐e55. 22074497 10.1111/j.1365-2982.2011.01806.x PMC3245326

[10] Rattan S . The internal anal sphincter: regulation of smooth muscle tone and relaxation. Neurogastroenterol Motil. 2005;17:50‐59. 15836455 10.1111/j.1365-2982.2005.00659.x

[11] Cobine CA , Fong M , Hamilton R , Keef KD . Species dependent differences in the actions of sympathetic nerves and noradrenaline in the internal anal sphincter. Neurogastroenterol Motil. 2007;19:937‐945. 17973631 10.1111/j.1365-2982.2007.00982.x

[12] Qu Z‐D , Thacker M , Castelucci P , Bagyánszki M , Epstein ML , Furness JB . Immunohistochemical analysis of neuron types in the mouse small intestine. Cell Tissue Res. 2008;334:147‐161. 18855018 10.1007/s00441-008-0684-7

[13] Sang Q , Young HM . Chemical coding of neurons in the myenteric plexus and external muscle of the small and large intestine of the mouse. Cell Tissue Res. 1996;284:39‐53. 8601295 10.1007/s004410050565

[14] Perez‐Medina AL , Galligan JJ . Immunohistochemical Identification of Purinergic Neurons in the Enteric Nervous System. FASEB J. 2017;31:1045‐1047.

[15] Opazo A , Lecea B , Gil V , Jiménez M , Clavé P , Gallego D . Specific and complementary roles for nitric oxide and ATP in the inhibitory motor pathways to rat internal anal sphincter. Neurogastroenterol Motil. 2011;23:e11‐e25. 20939852 10.1111/j.1365-2982.2010.01602.x

[16] Wade PR , Cowen T . Neurodegeneration: a key factor in the ageing gut. Neurogastroenterol Motil. 2004;16:19‐23. 15065999 10.1111/j.1743-3150.2004.00469.x

[17] Saffrey MJ . Ageing of the enteric nervous system. Mech Ageing Dev. 2004;125:899‐906. 15563936 10.1016/j.mad.2004.09.003

[18] Saffrey MJ . Aging of the mammalian gastrointestinal tract: a complex organ system. AGE. 2013;36:1019‐1032. 10.1007/s11357-013-9603-2 PMC4082571 24352567

[19] Wang C , Houghton MJ , Gamage PPKM , et al. Changes in the innervation of the mouse internal anal sphincter during aging. Neurogastroenterol Motil. 2013;25:e469‐e477. 23634828 10.1111/nmo.12144

[20] Lewicky‐Gaupp C , Hamilton Q , Ashton‐Miller J , Huebner M , DeLancey JOL , Fenner DE . Anal sphincter structure and function relationships in aging and fecal incontinence. Am J Obst Gynecol. 2009; 200:559.e1‐559.e5. 19136096 10.1016/j.ajog.2008.11.009 PMC3040636

[21] Huebner M , Margulies RU , Fenner DE , Ashton‐Miller JA , Bitar KN , DeLancey JOL . Age effects on internal anal sphincter thickness and diameter in nulliparous females. Dis Colon Rectum. 2007;50:1405‐1411. 17665265 10.1007/s10350-006-0877-7 PMC2288793

[22] Nielsen MB , Pedersen JF . Changes in the anal sphincter with age: an endosonographic study. Acta Radiol. 1996;37:357‐361. 8845269 10.1177/02841851960371P175

[23] Patel BA . Electroanalytical approaches to study signaling mechanisms in the gastrointestinal tract. Neurogastroenterol Motil. 2011;23:595‐605. 21481101 10.1111/j.1365-2982.2011.01708.x

[24] Patel BA , Arundell M , Parker KH , Yeoman MS , O'Hare D . Detection of nitric oxide release from single neurons in the pond snail, *Lymnaea stagnalis* . Analyt Chem. 2006;78:7643‐7648. 17105154 10.1021/ac060863w

[25] Patel BA , Patel N , Fidalgo S , et al. Impaired colonic motility and reduction in tachykinin signalling in the aged mouse. Exp Gerontol. 2014;53:24‐30. 24560671 10.1016/j.exger.2014.02.007

[26] Gamage PPKM , Ranson RN , Patel BA , Yeoman MS , Saffrey MJ . Myenteric neuron numbers are maintained in aging mouse distal colon. Neurogastroenterol Motil. 2013;25:e495‐e505. 23517051 10.1111/nmo.12114

[27] Patel BA , Dai X , Burda JE , et al. Inhibitory neuromuscular transmission to ileal longitudinal muscle predominates in neonatal guinea pigs. Neurogastroenterol Motil. 2010;22:909‐e237. 20482699 10.1111/j.1365-2982.2010.01508.x PMC2911488

[28] Yeoman M , Scutt G , Faragher R . Insights into CNS ageing from animal models of senescence. Nat Rev Neurosci. 2012;13:435‐445. 22595787 10.1038/nrn3230

[29] Perry E , Martin‐Ruiz C , Lee M , et al. Nicotinic receptor subtypes in human brain ageing, Alzheimer and Lewy body diseases. Eur J Pharmacol. 2000;393:215‐222. 10771016 10.1016/s0014-2999(00)00064-9

[30] Tribollet E , Bertrand D , Marguerat A , Raggenbass M . Comparative distribution of nicotinic receptor subtypes during development, adulthood and aging: an autoradiographic study in the rat brain. Neuroscience. 2004;124:405‐420. 14980390 10.1016/j.neuroscience.2003.09.028

[31] Burnstock G . Do some nerve cells release more than one transmitter? Neuroscience. 1976;1:239‐248. 11370511 10.1016/0306-4522(76)90054-3

[32] Mañé N , Gil V , Martínez‐Cutillas M , Clavé P , Gallego D , Jiménez M . Differential functional role of purinergic and nitrergic inhibitory cotransmitters in human colonic relaxation. Acta Physiol. 2014;212:293‐305. 10.1111/apha.12408 25327170

[33] Mañé N , Viais R , Martínez‐Cutillas M , Gallego D , Correia‐de‐Sá P , Jiménez M . Inverse gradient of nitrergic and purinergic inhibitory cotransmission in the mouse colon. Acta Physiol. 2016;216:120‐131. 10.1111/apha.12599 26347033

[34] Singh J , Kumar S , Krishna CV , Rattan S . Aging‐associated oxidative stress leads to decrease in IAS tone via RhoA/ROCK downregulation. Am J Physiol Gastrointest Liver Physiol. 2014;306:G983‐G991. 24742984 10.1152/ajpgi.00087.2014 PMC4042111

[35] Saffrey MJ . Cellular changes in the enteric nervous system during ageing. Dev Biol. 2013;382:344‐355. 23537898 10.1016/j.ydbio.2013.03.015

[36] Cirilo CP , Schoffen JPF , de Santi‐Rampazzo AP , et al. Dietary restriction interferes with oxidative status and intrinsic intestinal innervation in aging rats. Nutrition. 2013;29:673‐680. 23317927 10.1016/j.nut.2012.09.004

[37] Pascua P , Camello‐Almaraz C , Camello PJ , et al. Melatonin, and to a lesser extent growth hormone, restores colonic smooth muscle physiology in old rats. J Pineal Res. 2011;51:405‐415. 21649718 10.1111/j.1600-079X.2011.00904.x

[38] Pozo MJ , Gomez‐Pinilla PJ , Camello‐Almaraz C , et al. Melatonin, a potential therapeutic agent for smooth muscle‐related pathological conditions and aging. Curr Med Chem. 2010;17:4150‐4165. 20939818 10.2174/092986710793348536

[39] Wang C , Jurk D , Maddick M , Nelson G , Martin‐Ruiz C , Von Zglinicki T . DNA damage response and cellular senescence in tissues of aging mice. Aging Cell. 2009;8:311‐323. 19627270 10.1111/j.1474-9726.2009.00481.x

[40] Hetz S , Acikgoez A , Moll C , et al. Age‐related gene expression analysis in enteric ganglia of human colon after laser microdissection. Front Aging Neurosci. 2014;6:276. 25360110 10.3389/fnagi.2014.00276 PMC4197768

[41] O'Malley BW , Mengel CE , Meriwether WD , Zirkle LG . Inhibition of erythrocyte acetylcholinesterase by peroxides*. Biochemistry. 1966;5:40‐45. 5938954 10.1021/bi00865a006

[42] Méndez‐Garrido A , Hernández‐Rodríguez M , Zamorano‐Ulloa R , et al. In vitro effect of H2O2, some transition metals and hydroxyl radical produced via fenton and fenton‐like reactions, on the catalytic activity of AChE and the hydrolysis of ACh. Neurochem Res. 2014;39:2093‐2104. 25096900 10.1007/s11064-014-1400-5

[43] Liu H , Wu J , Yao J‐Y , Wang H , Li S‐T . The role of oxidative stress in decreased acetylcholinesterase activity at the neuromuscular junction of the diaphragm during sepsis. Oxid Med Cell Longev. 2017;2017:6. 10.1155/2017/9718615 PMC5694580 29230271

[44] Singh J , Kumar S , Rattan S . Bimodal effect of oxidative stress in internal anal sphincter smooth muscle. Am J Physiol Gastrointest Liver Physiol. 2015;309:G292‐G300. 26138467 10.1152/ajpgi.00125.2015 PMC4556951

[45] Rubio‐Ruiz ME , Pérez‐Torres I , Soto ME , Pastelín G , Guarner‐Lans V . Aging in blood vessels. Medicinal agents FOR systemic arterial hypertension in the elderly. Ageing Res Rev. 2014;18:132‐147. 25311590 10.1016/j.arr.2014.10.001

[46] Heredia DJ , Grainger N , McCann CJ , Smith TK . Insights from a novel model of slow‐transit constipation generated by partial outlet obstruction in the murine large intestine. Am J Physiol Gastrointest Liver Physiol. 2012;303:G1004‐G1016. 22961801 10.1152/ajpgi.00238.2012 PMC3517665

[47] Patel BA , Fidalgo S , Wang C , et al. The TNF‐α antagonist etanercept reverses age‐related decreases in colonic SERT expression and faecal output in mice. Sci Rep. 2017;7:42754. 28198447 10.1038/srep42754 PMC5309893

